# Immunotherapy for Upper Tract Urothelial Carcinomas: A 2026 Update

**DOI:** 10.3390/cancers18101643

**Published:** 2026-05-19

**Authors:** Ilias Giannakodimos, Afroditi Ziogou, Foteini Grigoriou, Konstantinos Evmorfopoulos, Ioannis Zachos, Dimitrios Deligiannis, Zisis Kratiras, Vasileios Tzortzis, Aristotelis Bamias, Michael Chrisofos, Nikolaos Charalampakis

**Affiliations:** 1 Third Department of Urology, School of Medicine, Attikon University Hospital, National and Kapodistrian University of Athens, 12462 Athens, Greece; 2 Department of Internal Medicine, 417 Army Equity Fund Hospital, 11521 Athens, Greece; 3 Second Propaedeutic Department of Internal Medicine, Attikon University Hospital, National & Kapodistrian University of Athens, 12462 Athens, Greece; 4 Department of Urology, Faculty of Medicine, School of Health Sciences, University Hospital of Larissa, University of Thessaly, 41100 Larissa, Greecejohnbzac@yahoo.gr (I.Z.);; 5 Department of Medical Oncology, Metaxa Cancer Hospital of Piraeus, 18537 Piraeus, Greece

**Keywords:** urothelial cancer, upper tract urothelial carcinoma, upper urinary tract, immunotherapy, systematic therapy

## Abstract

Upper tract urothelial carcinoma (UTUC) accounts for approximately 5–10% of diagnosed urothelial carcinoma (UC). Due to advanced initial presentation or aggressive progression of UTUC, the majority of patients will need systematic treatment in the neo-adjuvant or adjuvant setting. The management of patients with UTUC is complicated due to the lack of published data and only limited studies addressing immunotherapy in UTUC patients are available. The perioperative scenario in localized high-risk disease is still evolving. For metastatic disease, two monoclonal antibodies targeting PD-1 (pembrolizumab and nivolumab) and three to its ligand PD-L1 (atezolizumab, avelumab and durvalumab) have obtained approval for the second-line treatment of platinum-pretreated patients. Atezolizumab and pembrolizumab are currently approved in the first-line setting for cisplatin ineligible patients, with PD-L1- positive tumors. The aim of this review was to highlight the role of immunotherapy in locally advanced and metastatic UTUC and provide the safety profile of these regimens.

## Simple Summary

Upper tract urothelial carcinoma constitutes a rare and aggressive malignancy regarding which evidence regarding immunotherapy remains limited. Most available data derive from studies on urothelial carcinoma of the bladder, while patients with upper tract disease are underrepresented. This review summarizes current evidence on the use of immunotherapy in localized, locally advanced as well as metastatic upper tract urothelial carcinoma, focusing on therapeutic efficacy and safety. In addition, it highlights current challenges and gaps in the literature. The findings of this review may support clinical decision-making and promote further research dedicated specifically to upper tract urothelial carcinoma, aiming to improve evidence-based management and patient outcomes.

## 1. Introduction

Upper tract urothelial carcinoma (UTUC) represents a relatively uncommon subtype of urothelial carcinoma, comprising nearly 5–10% of all diagnosed cases; almost 50% of affected patients present with synchronous bladder urothelial carcinoma (UCB) [1]. In Western populations, the incidence of UTUC is estimated at approximately two cases per 100,000 individuals annually, predominantly affecting elderly patients. In contrast, several Asian countries report a substantially higher prevalence, with UTUC accounting for up to one quarter of all urothelial carcinomas and often demonstrating a more aggressive clinical behavior [2]. The current standard therapeutic approach includes radical nephroureterectomy accompanied by lymph node dissection and excision of the bladder cuff. Furthermore, the incorporation of perioperative platinum-based combination chemotherapy has been associated with superior oncological outcomes compared with surgery alone and is currently recommended by international guidelines for patients with locally advanced disease [3,4].

Interestingly UTUC may initially present with aggressive clinical presentation; more than half of patients are found to have muscle-invasive disease, which is often already metastatic [5]. Due to advanced initial presentation or aggressive progression of UTUC, the majority of patients will need systematic treatment in the neo-adjuvant or adjuvant setting. Although the advantage of platinum combination treatment is well described in these patients and comprises the first line treatment, the majority of these patients are unable to receive platinum-based chemotherapy due to impaired renal function. In 2016, the US Food and Drug Administration (FDA) approved the first drug for the systemic immunotherapy treatment of UC patients [6]. From this date, several phase 2 and 3 clinical trials were developed aiming to establish the role of immunotherapy in these patients. More recently, the therapeutic landscape of advanced urothelial carcinoma has rapidly evolved, with the combination of enfortumab vedotin and pembrolizumab emerging as a central first-line treatment strategy in metastatic disease. In parallel, perioperative immunotherapy approaches are increasingly being investigated in high-risk localized disease, both in the neo-adjuvant and adjuvant setting. Although different immunotherapy agents have been approved for both neo-adjuvant and adjuvant treatment in metastatic BC, their role in UTUC patients has not been established yet, since no RCTs have been designed for this specific population. Their role as first-line or second-line treatment in adjuvant therapy or as maintenance treatment is promising, but still further well-designed trials are needed focusing on this subset of patients [7].

The aim of this review was to update the current knowledge on the role of immunotherapy in locally advanced and metastatic UTUC, to summarize published and ongoing trials in this specific population, while also highlight the safety profile of these regimens.

## 2. Methods

This manuscript constitutes a narrative review of the current literature on immunotherapy in UTUC. A targeted search strategy was used until 15 April 2025 to identify relevant studies, with emphasis on pivotal clinical trials and evidence informing contemporary clinical practice. Priority was given to phase III randomized controlled trials, supplemented by key phase II studies when phase III data were not available. In addition, landmark studies that have contributed to current international guideline recommendations were included, as well as selected ongoing or recently reported trials providing insight into emerging therapeutic strategies. No strict time restriction was applied; however, preference was given to contemporary evidence. Study selection was based on clinical relevance to UTUC and its management across metastatic and perioperative settings.

## 3. Neo-Adjuvant Immunotherapy

The survival benefit and oncologic outcomes of administering platinum-based chemotherapy prior to surgery to patients with muscle-invasive bladder cancer (MIBC) has already been established [8]. The main advantage of neo-adjuvant chemotherapy concerns patients with optimal renal function that receive cisplatin-based regimens. Although prospective data and ongoing trials are promising, no RCT has been published to establish the role of neo-adjuvant chemotherapy in UTUC. Of note, up to 50% of candidates are proved unfit for cisplatin-based chemotherapy and thus, other regimens should be considered in the neoadjuvant setting (Table S1) [8].

As far as immunotherapy is concerned, only a small phase II study, including 10 UTUC patients with high-risk features, has evaluated the role of pembrolizumab in the neoadjuvant treatment. More specifically, in PURE-02 trial, after administration of 3 courses of pembrolizumab, one treatment-related death occurred, one patient had complete clinical response, two presented with disease progression, while the remaining patients remained non-responders [9]. As a result, currently there is no evidence to support the use of neoadjuvant immunotherapy in high-risk UTUC and further well-designed multicenter RCTs are needed to evaluate its role [1].

## 4. Adjuvant Immunotherapy for Non-Metastatic UTUC

Patients diagnosed with high-risk localized upper tract urothelial carcinoma are generally encouraged to undergo radical nephroureterectomy combined with excision of the bladder cuff and regional lymph node dissection [1]. In this population, the use of adjuvant platinum-based chemotherapy is highly recommended and supported by level I evidence [1]. Strong evidence for this therapeutic approach derives from the phase III POUT trial, which showed that postoperative platinum-based chemotherapy after radical nephroureterectomy significantly prolonged disease-free survival compared with observation alone, thereby establishing adjuvant chemotherapy as the current standard treatment strategy in this clinical setting [10]. Numerous immunotherapy strategies, whether as single agents or in combination, along with chemo-immunotherapy protocols, have been evaluated in the adjuvant context. The efficacy rates are not equal to those achieved with cisplatin-based regimens, presenting with substantial adverse effects, especially with dual immune checkpoint inhibitor regimens, while comprehensive overall survival (OS) data and prognostic biomarkers remain insufficient [7]. Interesting, these trials include only a restricted cohort of UTUC patients and findings mainly derive from extrapolation of data.

The IMvigor 010 trial involved the randomization of 809 high-risk UC patients being administered either adjuvant atezolizumab or placebo, with merely 7% of the atezolizumab and 6% of the placebo arm affected by UTUC [11]. After 19.4 months, no difference in median DFS was observed between the study groups [11]. Additionally, the Checkmate 274 trial, a phase III, multicentre RCT, included 709 high-risk muscle-invasive UC, of whom 21% were identified with UTUC, and aimed to evaluate the adjuvant use of nivolumab [12]. This trial yielded favorable outcomes, resulting in FDA’s and EMA’s approval of the medication, since the nivolumab group demonstrated a median DFS of 20.8 months, compared to the 10.8 months recorded for the placebo group. However, on subgroup analysis, no significant benefit was found in UTUC patients after administration of adjuvant nivolumab, requiring further follow-up and analysis [12]. Importantly, the updated 5-year follow-up analysis demonstrated that nivolumab was associated with longer median DFS compared with placebo among all randomized patients (21.9 versus 11.0 months; hazard ratio 0.74, 95% confidence interval 0.61–0.90). Median OS also appeared improved in the nivolumab group, reaching 75.0 months compared with 50.1 months in the placebo arm (hazard ratio 0.83, 95% confidence interval 0.67–1.02). However, subgroup analysis did not reveal a statistically significant benefit in either DFS or OSamong patients with upper tract urothelial carcinoma [13].

In another phase III trial, 702 MIBC patients with high-risk features treated with either radical cystectomy or RNU, were randomly assigned to receive either adjuvant pembrolizumab or observation [14]. The median DFS was significantly longer with pembrolizumab (29.6 months) compared to observation (14.2 months) and adverse events >Grade 3 occurred in 50.6% of the patients in the pembrolizumab group. However, the proportion of UTUC patients (25% of overall population) in the study was small and on subgroup analyses, no significant benefit was reported from adjuvant pembrolizumab [14]. Finally, in a network meta-analysis of different adjuvant treatments for UC patients, nine studies with 2444 patients were included. Adjuvant platinum-based chemotherapy had superior oncologic outcomes compared to ICI for UTUC patients, while the incidence ratio of adverse events was similar between groups [15]. Published and ongoing trials regarding adjuvant immunotherapy for non-metastatic UTUC are shown in Table 1.

## 5. Perioperative Immunotherapy

Perioperative immunotherapy has emerged as a promising strategy in urothelial carcinoma (UC), aiming to improve surgical outcomes through the integration of systemic therapy before and after resection.

In cisplatin-ineligible patients with muscle-invasive bladder cancer (MIBC), the phase III KEYNOTE-905/EV-303 trial evaluated enfortumab vedotin (EV), an anti–Nectin-4 antibody–drug conjugate, in combination with pembrolizumab, an anti–PD-1 agent. The combination demonstrated a substantial clinical benefit compared with surgery alone, with a 60% reduction in the risk of recurrence, progression, or death (hazard ratio [HR] 0.40) and a 50% reduction in the risk of death (HR 0.50). Pathological complete response (pCR) rates were significantly higher in the combination arm (57.1% vs. 8.6%) [16].

In cisplatin-eligible patients, the phase III KEYNOTE-B15/EV-304 trial (NCT04700124) further supported the efficacy of EV plus pembrolizumab in the perioperative setting compared with standard neoadjuvant gemcitabine–cisplatin chemotherapy. The combination reduced the risk of recurrence, progression, or death by 47% (HR 0.53) and achieved significantly higher pCR rates (55.8% vs. 32.5%). At the time of analysis, median event-free survival (EFS) and overall survival (OS) had not yet been reached, suggesting a durable clinical benefit. The safety profile was consistent with previous reports, with no new safety signals identified. This regimen is administered both preoperatively (neoadjuvant) and postoperatively (adjuvant), and is emerging as a potential new standard of care for cisplatin-eligible patients [17].

Another important advance comes from the phase III NIAGARA trial, which evaluated perioperative durvalumab in combination with neoadjuvant gemcitabine and cisplatin followed by radical cystectomy and adjuvant durvalumab. The addition of durvalumab resulted in a 32% reduction in the risk of recurrence, progression or death (HR for EFS 0.68) and significantly improved overall survival compared with chemotherapy alone. The regimen also increased pCR rates by approximately 10%. Importantly, the incorporation of durvalumab did not increase perioperative morbidity or surgical complications, and the treatment was well tolerated. Based on these findings, this approach has been established as a new standard of care and durvalumab has received regulatory approval in this setting [18].

In parallel, the phase III KEYNOTE-866 trial is investigating the addition of pembrolizumab to standard cisplatin-based neoadjuvant chemotherapy followed by adjuvant pembrolizumab. Although mature survival data are still pending, early results indicate improved pathological response rates and a potential benefit in event-free survival with the incorporation of immune checkpoint inhibition into standard perioperative chemotherapy [16]. Although these trials focus on bladder cancer, their findings are of particular relevance to UTUC, where prospective perioperative immunotherapy data remain limited and current treatment strategies are largely extrapolated from MIBC studies.

Published and ongoing trials regarding perioperative immunotherapy for non-metastatic UTUC are shown in Table 2.

## 6. Immunotherapy for Metastatic UTUC (First and Second Line Treatment)

### First-Line Systemic Therapy

A major breakthrough in the management of metastatic UTUC occurred with the introduction of EV, in combination with pembrolizumab. Early-phase data from EV-103 (cohort A),a phase Ib/II trial evaluating EV plus pembrolizumab as first-line therapy in cisplatin-ineligible patients, demonstrated an impressive overall response rate of 73% and durable responses, leading to accelerated FDA approval of the regimen [19]. The confirmatory phase III EV-302/KEYNOTE-A39trial compared EV plus pembrolizumab with platinum–gemcitabine chemotherapy in patients with previously untreated locally advanced or metastatic urothelial carcinoma, showing significant improvements in progression-free survival (PFS; 12.5 vs. 6.3 months, HR 0.45) and overall survival (OS; 31.5 vs. 16.1 months, HR 0.47). Patients with UTUC comprised approximately 27% of the study population, and no significant difference was observed based on tumor site (PFS HR ~0.50; OS HR ~0.53). These findings established EV plus pembrolizumab as the new global standard of care for previously untreated locally advanced or metastatic UC [20]. For patients who are ineligible for EV, chemo-immunotherapy remains an alternative first-line option. In cisplatin-eligible patients, platinum-based chemotherapy followed by avelumab maintenance remains an established treatment pathway supported by level I evidence [1].

TheCheckMate-901phase III trial compared nivolumab plus gemcitabine–cisplatin versus chemotherapy alone and the combined therapy improved median OS: 21.7 vs. 18.9 months; HR 0.78 and PFS: HR 0.72 [21]. Responses were consistent across subgroups, including UTUC accounted for 18.4% of patients in the nivolumab arm and 25.3% in the GC arm. Interestingly, UTUC-specific outcomes were reported in an Asian subgroup analysis of CheckMate-901, in which UTUC accounted for 27.8% of the nivolumab plus gemcitabine–cisplatin arm and 36.1% of the gemcitabine–cisplatin arm [22]. The median OS was 23.9 months with nivolumab plus gemcitabine–cisplatin and 21.7 months with gemcitabine–cisplatin alone in patients with BC (HR, 0.77 [95% CI, 0.43–1.40]) [21]. Of note, only in UTUC patients, median OS was estimated at 28.9 months with nivolumab plus gemcitabine/cisplatin against 15.3 months only with nivolumab (HR, 0.66 [95% CI, 0.29–1.53]) [22]. Collectively, these results suggest that the addition of nivolumab into first-line cisplatin-based chemotherapy provides clinically meaningful benefit both for metastatic BC and UTUC patients, but UTUC population was small and these findings cannot establish definitive superiority. Recently, the combination of ICI with chemotherapy for first-line treatment of adult patients with unresectable or metastatic urothelial carcinoma, based on CheckMate-901, has received both FDA and EMA approval [1].

Alternative first-line chemoimmunotherapy combinations were examined in JAVELIN trial (NCT03317496), a phase Ib/II study, evaluating avelumab plus chemotherapy in metastatic UC, including UTUC. Although ORRs were acceptable, there was no clear additive benefit to standard platinum–gemcitabine alone [23]. In addition, in the phase III KEYNOTE-361 trial, pembrolizumab was administered alone or in combination with platinum-based chemotherapy versus chemotherapy alone in previously untreated advanced UC, with 21% of UCs to be found in UUT. Of note, the study did not meet its co-primary endpoints, failing to demonstrate a significant improvement in PFS (8.3 vs. 7.1 months; HR 0.78) and OS (17.0 vs. 14.3 months; HR 0.86), while pembrolizumab monotherapy showed no OS benefit in exploratory analyses [24].

Patients with UTUC are frequently classified as cisplatin-ineligible due to impaired renal function, which may arise from tumor-related obstruction or from the loss of renal mass following nephrectomy. Consequently, this population is at increased risk for chemotherapy-induced nephrotoxicity [25]. In these patients, atezolizumab and pembrolizumab have demonstrated clinically meaningful activity as single agents. In Cohort 1 of the phase II, single-arm IMvigor210 trial, treatment-naive patients with locally advanced or metastatic UC who were ineligible for cisplatin-containing chemotherapy received atezolizumab. In the final long-term analysis, the ORR was estimated at 23.5% (95% CI 16.2–32.2%) in the overall population and at 28.1% (95% CI 13.8–46.8%) in the PD-L1 IC2/3 subgroup. The 5-year OS rate was 21.6% (95% CI 13.7–29.5%) for all patients and 27.0% (95% CI 10.3–43.7%) for the IC2/3 subgroup. Patients with UTUC accounted for approximately 28% of the cohort (*n* = 33). In an early interim analysis, the UTUC subgroup showed an ORR of 42.4%; however, the final analysis did not report UTUC-specific efficacy or survival outcomes [26]. However, the confirmatory phase III IMvigor130 trial did not demonstrate a survival benefit and thus, the first-line indication for cisplatin-ineligible patients was withdrawn [27].

Similarly, in KEYNOTE-052, a phase II, single-arm trial, treatment-naïve cisplatin-ineligible patients with locally advanced or metastatic UC received pembrolizumab monotherapy. In an early interim analysis, at a median follow-up of approximately 5 months, the ORR was 24% in the overall population and 38% among patients with a PD-L1 combined positive score (CPS) ≥ 10. The study included approximately 19% UTUC patients, who demonstrated an ORR of 22%, compared with 28% in those with lower UT. As a result, pembrolizumab initially received accelerated approval by the FDA and EMA for first-line treatment of cisplatin-ineligible patients with advanced UC. With extended follow-up to a minimum of 2 years, the confirmed ORR increased to 28.6% (95% CI 24.1–33.5%), while median overall survival (OS) was estimated at 11.3 months (95% CI 9.7–13.1 months) [28]. Among patients with PD-L1 expression, the ORR was 47.3% (95% CI 37.7–57.0%) and median OS was 18.5 months (95% CI 12.2–28.5 months). In the UTUC subgroup analysis, the ORR was 26.1%, compared with 29.3% in patients with lower-tract tumors, while median OS was estimated at 10.8months (95% CI 7.6–17.0 months) compared to 11.5 months (95% CI 9.7–13.1 months), respectively [28]. Following subsequent regulatory review and evaluating the results of the phase III KEYNOTE-361 trial, this indication was refined and pembrolizumab monotherapy was approved for cisplatin-ineligible adults with locally advanced or metastatic urothelial carcinoma whose tumors express PD-L1 with CPS ≥ 10 [29]. In addition, durvalumab was initially evaluated in the first-line setting for metastatic UC in the phase III DANUBE trial, which investigated durvalumab alone or in combination with tremelimumab (PD-1/CTLA-4 Inhibitor) compared with platinum-based chemotherapy. Interestingly, UTUC accounted for approximately 21% of enrolled patients [30]. Although the study did not meet its primary OS endpoints, exploratory analysis suggested potential benefit for the durvalumab/tremelimumab combination in patients with high PD-L1 expression (≥25%) [31].

Molecularly guided sequencing is increasingly emerging as a relevant consideration in metastatic UTUC, particularly in the context of biomarker-driven treatment selection beyond PD-L1. HER2 alterations, although less common in UTUC compared with other tumor types, are being explored as potential actionable targets, with early-phase data suggesting therapeutic activity of HER2-directed strategies in selected UTC subgroups [32]. In parallel, circulating tumor DNA (ctDNA) is gaining attention as a minimally invasive tool for real-time molecular profiling, treatment monitoring as well as detection of minimal residual disease. Integrating ctDNA dynamics with therapeutic decision-making may help refine sequencing strategies after first-line immunotherapy-based regimens, enabling earlier identification of resistance and facilitating transition to targeted systemic therapies [33]. Collectively, these biomarkers highlight a shift toward precision-guided sequencing approaches in metastatic UTUC rather than established treatment algorithms.

Published and ongoing trials regarding immunotherapy in the first line setting are shown in Table 3.

## 7. Maintenance Immunotherapy

In the phase III JAVELIN Bladder 100 trial, maintenance therapy with avelumab after disease control with first-line platinum-based chemotherapy established the role of immunotherapy maintenance in advanced or metastatic UC. Patients with no progression after four to six cycles of platinum-based chemotherapy were randomized to receive avelumab plus best supportive care (BSC) versus BSC alone.In the overall population, maintenance therapy with avelumab significantly prolonged OS compared to BSC (median OS 21.4 vs. 14.3 months; HR: 0.69; 95% CI 0.56–0.86) and improved median PFS (3.7 vs. 2.0 months; HR: 0.62) [29].UTUC patients comprised approximately 26.7% (187/700) of the included population [31]. In an exploratory subgroup analyses the relative benefit was smaller for upper-tract tumors than for lower-tract tumors (UTUC: OS HR 0.90, 95% CI 0.59–1.39; PFS HR 0.85, 95% CI 0.60–1.21). Due to lower statistical power of subgroup analyses, benefit from immunotherapy maintenance in this subgroup remains uncertain and additional studies are needed for UTUC population [34].

There are many ongoing trials exploring maintenance combinations. The ongoing JAVELIN Bladder Medley umbrella trial aims to explore multiple avelumab-based maintenance combinations after first-line platinum-based chemotherapy [29]. In its interim analysis, although avelumab plus sacituzumabgovitecan (SG) yielded longer PFS (11.2 vs. 3.8 months; HR 0.49) compared to avelumab alone, this benefit was accompanied by substantially higher rates of grade ≥3 toxicity (70% vs. 0%). In this trial, UTUC-specific results are awaited [35,36]. Published and ongoing trials regarding immunotherapy in the maintenance setting are summarized in Table 3.

## 8. Second Line Systemic Therapy

Concerning patients with metastatic or locally advanced/unresectable UC that have recurred or progressed following platinum-based chemotherapy, pembrolizumab constitutes the only ICI that has demonstrated OS benefit in a phase III trial. As already mentioned, evidence specific for UTUC cases is derived predominantly from subgroup analyses, or extrapolation, since no RCT has focused exclusively on this population. KEYNOTE-045 is a phase 3 trial that randomized participants to receive either paclitaxel, docetaxel, vinflunine or pembrolizumab in the post-platinum setting. Pembrolizumab demonstrated improved OS in the overall population versus chemotherapy (10.3 vs. 7.4 months; HR 0.73) and presented with a better safety profile and durable responses across all PD-L1 subgroup [37]. Only a small proportion of enrolled patients included UTUC cases (approximately 14%) and an exploratory subgroup analysis suggested a lower OS hazard ratio for these patients (HR 0.53) compared with tumors of the lower urinary tract (HR 0.77) [37].

Additionally, according to CheckMate-275, a phase II, single arm clinical trial, nivolumab was also evaluated in the second line setting of unresectable or metastatic UC, in which patients received nivolumab after progression or recurrence following platinum-based chemotherapy. Monotherapy with nivolumab achieved an ORR of 19.6% and was associated with a median OS of 8.74 months in the total population, showing durable responses regardless of PD-L1 expression. Although UTUC patients were included, no separate subgroup analysis for these patients was reported [38]. Based on this phase II study, nivolumab received regulatory approval as second-line monotherapy after prior platinum therapy in metastatic or unresectable UC. However, no randomized phase III trial has been conducted to confirm these findings.

Atezolizumab was also assessed as monotherapy for patients with locally advanced or metastatic UC who have progressed during or following a prior platinum-based chemotherapy regimen, in the Phase II IMvigor 210 trial.Of note, ORRs were 26% in the PDL1 > 5% group, and 15% in the overall population. At the final analysis, median OS was estimated at 7.9 months in all patients and at 11.9 months in the IC2/3 subgroup [39]. Interestingly, durable responses included UTUC patients (21% of the overall population) and patients with poor prognostic features [26,39]. Based on these results atezolizumab was initially granted accelerated/conditional approval as a second line regimen after platinum-based chemotherapy, but the confirmatory IMvigor211 trial did not meet its primary OS endpoint [40,41]. Published and ongoing trials regarding immunotherapy in the second line and maintenance setting are presented in Table 4.

## 9. Proposed Clinical Positioning of Immunotherapy in UTUC

Immunotherapy in UTUC should be individualized according to disease stage, treatment setting and patient eligibility. In metastatic disease, ICI–based combinations represent the backbone of first-line therapy, with enfortumab vedotin plus pembrolizumab currently considered the preferred standard of care. Chemo-immunotherapy may be used in selected cisplatin-eligible patients, while immunotherapy-based approaches remain an option in cisplatin-ineligible or PD-L1–selected cases. Maintenance avelumab after response to platinum-based chemotherapy is an established strategy, although subgroup data suggest a less pronounced benefit in UTUC. In the second-line setting, pembrolizumab is the preferred immune checkpoint inhibitor based on overall survival benefit, with nivolumab and atezolizumab as alternative options.

In contrast, perioperative immunotherapy remains investigational in UTUC. Neoadjuvant strategies are limited to early-phase studies, and adjuvant benefit is not consistently demonstrated in UTUC subgroups; thus, platinum-based chemotherapy remains standard. Overall, immunotherapy is established in metastatic disease, whereas perioperative use remains exploratory.

## 10. Safety Profile

Immunotherapy has become an increasingly important therapeutic option for patients with UTUC, particularly in the advanced or metastatic setting, with immune checkpoint inhibitors (ICIs) representing the most established approach [42]. As already mentioned, dedicated trials for UTUC are limited; the available safety data largely derive from broader UC studies that include UTUC patients as subgroups. This data consistently indicates that tolerability profiles of ICIs in UTUC reflect the ones observed in BC [43,44]. Overall, ICIs are linked to a manageable spectrum of immune-related adverse events and are generally better tolerated than conventional platinum-based chemotherapy [45].

The toxicities related to ICIs primarily arise from immune system activation, which can provoke inflammatory reactions in healthy tissues. These immune-related adverse events (irAEs) most frequently affect the skin, endocrine organs, gastrointestinal tract, liver and lungs [45,46]. Dermatologic reactions, including rash and pruritus, are the most common, but usually of mild intensity. Endocrine abnormalities, including thyroid dysfunction or, less frequently, adrenal insufficiency, are also typical and often require long-term hormone replacement [47]. Gastrointestinal adverse events, such as diarrhea and immune-mediated colitis, may require treatment interruption and corticosteroids depending on severity [48]. Hepatotoxicity and pneumonitis represent additional well-recognized inflammatory conditions. Most of these reactions are low-grade and reversible with timely intervention, although severe irAEs can occur in a minority of patients [47,49].

Notably, many patients with UTUC usually present with impaired renal function, either due to underlying hydronephrosis or as a consequence of nephroureterectomy. Unlike platinum-based chemotherapy, ICIs do not require dose adjustment for reduced renal function and exhibit minimal direct nephrotoxicity [50]. Immune-mediated nephritis is rare and typically resolves with immunosuppressive therapy. This renal safety advantage contributes to the suitability of ICIs for UTUC patients who are often elderly or medically frail and may be ineligible for cisplatin [51,52]. Regarding other immunotherapies, such as BCG or investigational tumor vaccines, systemic nephrotoxicity is generally minimal; local reactions and procedure-related risks may occur, highlighting a complementary safety profile to ICIs [53,54].

A compelling demonstration of the favorable safety profile of immunotherapy in UTUC can be derived from large-scale trials in UC. Pembrolizumab was associated with a lower incidence of treatment-related adverse events of any grade, as well as a substantially reduced rate of severe (grade 3–5) toxicities, compared with chemotherapy. Moreover, discontinuations due to adverse events were less frequent in the immunotherapy arm [55]. These findings underscore that ICIs offer a more favorable risk–benefit profile than conventional chemotherapy, even in patients with UTUC, many of whom present with compromised renal function [56]. Although data are limited, early-phase studies of other immunotherapies, including BCG instillation and adoptive T-cell therapy, suggest manageable safety profiles. In a multicenter study of 162 patients with intermediate or high-risk NMBC treated with BCG, adverse events occurred in 14.8% of patients over a 3 year follow-up; the most common reported were hematuria and dysuria, all grade 1–2. No life-threatening events or sepsis were observed [57].

In summary, immunotherapy for UTUC is characterized by predictable immune-mediated toxicities that are typically manageable and occur at lower intensity compared with traditional chemotherapy. The absence of significant renal toxicity further enhances its applicability in UTUC, where kidney function is often compromised [58,59]. Evidence from major urothelial carcinoma trials, including those that enrolled UTUC patients, reinforces the view that the safety profile of ICIs is acceptable, clinically manageable and generally superior to that of systemic chemotherapy. A critical aspect is the timely recognition and accurate differential diagnosis of these events, as delays can exacerbate symptoms and increase the risk of complications [44]. As the role of immunotherapy expands into earlier disease settings, future studies focusing specifically on UTUC will aid in refining the understanding of its safety in this patient population.

## 11. Conclusions

Although several immunotherapeutic agents have been approved in the perioperative setting and in the first and second line treatment of UTUC patients with metastatic disease, their role mainly derives from extrapolation of data from trials designed for UC, mainly derived from the bladder. Further well-designed trials for patients with UTUC are needed to further elucidate the role of immunotherapy in the neoadjuvant and in the adjuvant setting, as well as in the first line and second line immunotherapy setting in metastatic UTUC.

## Figures and Tables

**Table 1:** Published and ongoing trials regarding adjuvant immunotherapy for non-metastatic UTUC.

Trial	Regimens	Line/Study Design	Total Patients/UTUC pts (%)	Outcomes in Total pts	Outcomes in UTUC pts
IMvigor010 trial [11]	Atezolizumab vs. observation	1 L/Phase 3	809–54 (6.7%)	DFS: 19.4 vs. 16.6 months HR: 0.89 (0.74–1.08)	DFS: 14.2 vs. 28.1 months HR: 1.25 (0.57–2.74)
Checkmate 274 NCT02632409 [12]	Nivolumab vs. Placebo	1 L/Phase 3	709–149 (21%)	DFS: 21.9 vs. 11 months HR: 0.74 (0.61–0.90) OS: 75 vs. 50.1 months HR: 0.83 (0.67–1.02)	DFS: renal-pelvis HR 1.24 (0.7–2.2) ureteral tumors HR 1.56 (0.70–3.48) OS: Renal HR: 1.12 (0.56–2.24) Ureter: HR 4.01 (1.22–13.2)
AMBASSADORNCT03244384 [14]	Pembrolizumab vs. observation	1 L/Phase 3	702–154 (21.9%)	DFS: 29.6 vs. 14.2 months HR: 0.73 (0.59 to 0.90)	DFS: HR: 1.27 (0.74–2.18)

DFS: disease-free survival, OS: Overall Survival, HR: Hazard ratio.

**Table 2:** Published and ongoing trials regarding perioperative immunotherapy for non-metastatic UTUC.

Trial Name	Regimen Used (Experimental vs. Control)	Phase	Total Patients	Outcomes in Total Patients	Outcomes in UTUC Patients
KEYNOTE-905/EV-303	Enfortumab vedotin + pembrolizumab → surgery → adjuvant therapy vs. surgery alone	Phase III	~344 randomized (reported interim cohort)	↓ Recurrence/progression/death by 60% (HR 0.40); ↓ death by 50% (HR 0.50); pCR: 57.1% vs. 8.6%	Not available (UTUC not included)
KEYNOTE-B15/EV-304	Enfortumab vedotin + pembrolizumab vs. neoadjuvant gemcitabine–cisplatin	Phase III	~886 planned	↓ Recurrence/progression/death by 47% (HR 0.53); pCR: 55.8% vs. 32.5%; EFS and OS not reached	Not available (UTUC not included)
NIAGARA	Durvalumab + neoadjuvant gemcitabine/cisplatin → surgery → adjuvant durvalumab vs. chemotherapy alone	Phase III	1063 patients	↓ EFS events by 32% (HR 0.68); improved OS; ~10% increase in pCR; no increase in perioperative complications	Not available (UTUC not included)
KEYNOTE-866	Pembrolizumab + neoadjuvant cisplatin-based chemotherapy → adjuvant pembrolizumab vs. chemotherapy alone	Phase III	~870 planned	Improved pathological response; trend toward improved EFS; OS immature data	Not available (UTUC not included)

EFS: event-free survival, OS: overall survival, HR: hazard ratio, pCR: pathological complete response.

**Table 3:** Published and ongoing trials regarding immunotherapy in the first line setting of UC.

Trial	Regimens	Line/Study Design	Total Patients/UTUC pts (%)	Outcomes in Total pts (Experimental vs. Control)	Outcomes in UTUC pts
NCT04223856 EV-302 [20]	EV + pembrolizumab vs. platinum-based chemotherapy	1 L/Phase 3	886/239 (27%)	PFS: 12.5 vs. 6.3 months, HR 0.45, 0.38 to 0.54 OS: 31.5 vs. 16.1 mo, HR 0.47, 0.38 to 0.58	PFS: 12.7 vs. 6.2 months HR: 0.50 (0.35–0.71) OS: (NE vs. 18.4 months HR: 0.53 (0.34–0.83)
NCT03036098 CheckMate 901 [21]	Nivolumab with gemcitabine/cisplatin vs. gemcitabine/cisplatin	1 L/phase 3	608–77 (13%)	PFS: 7.9 vs. 7.6 months HR: 0.72, 0.59–0.88 OS: 21.7 vs. 18.9 months HR 0.78, 0.63–0.96 ORR: 57.6% vs. 43.1%	NA
NCT02335424 KEYNOTE-052 [28]	Pembrolizumab in cisplatin-ineligible patients	1 L/phase 2	370/69 (19%)	ORR: 28.6–22% OS: 11.3 months (9.7 to 13.1) CPS ≥ 10, ORR: 47.3 (37.7–57.0%)	OS: 10.8 months (7.6 to 17.0 months) ORR: 26.1%
NCT02853305 KEYNOTE-361 [24]	Pembrolizumab plus chemotherapy vs. pembrolizumab alone vs. chemotherapy	1 L/phase 3	1010–211 (21%)	Pembro with chemo vs. chemo PFS: 8.3 vs. 7.1 months HR 0.78 (0.65–0.93) OS: 17.0 vs. 14.3 months HR 0.86 (0.72–1.02)	OS: 0.88 (0.59–1.31)
NCT02807636 IMvigor130 [27]	Atezolizumab plus platinum-based chemotherapy (group A), atezolizumab alone (group B), or placebo plus platinum-based chemotherapy (group C)	1 L/phase 3	1213–312 (25.7%)	OS: 16.1 group A vs. 13.4 months group C HR: 0.85 (0.73–1.00) OS: 15.2 group B vs. 13.3 months group C HR: 0.98 (0.82–1.16)	NA
NCT02516241 DANUBE trial [30]	Durvalumab (±tremelimumab) vs. chemotherapy	1 L/phase 3	1032–221 (~21.4%)	OS: 15.1 vs. 12·1 months HR: 0.85 (0.72–1.02)	NA
NCT02108652-NCT02951767 IMvigor210 [26]	Cohort 1: Treatment-naive Cisplatin Ineligible Participants/Cohort 2: participants who have progressed during or following a prior platinum-based chemotherapy regimen.	1 L, 2 L/Phase 2	cohort 1: *n* = 33?/cohort 2: 310–65 (21%)	OS: Cohort 1: 16.1 months/Cohort 2: 7.9 mo ORR: cohort 1: 23.5–42.4%/cohort 2: 15%	NA

PFS: progression-free survival, OS: overall survival, ORR: objective response rate, NA: Not Available.

**Table 4:** Published and ongoing trials regarding immunotherapy in the second line and maintenance setting of UC.

Trial	Regimen	Line	Total Patients	UTUC Patients	Outcomes in UTUC Patients
NCT02387996 (CheckMate 275) [38]	Nivolumab after at least one platinum-based chemotherapy	2L/phase 2	535	OS: 8.74 mo ORR: 19.6%	NA
NCT02302807 (IMvigor211) [41]	Atezolizumab vs. chemotherapy following a platinum-containing regimen.	2L/Phase 3	NA	OS: 8.6 vs. 8.0 months HR: 0.87 (0.71–0.94)	NA
NCT02256436 (KEYNOTE-045) [37]	Pembrolizumab vs. chemo (investigator choice)	2L/Phase 3	542–75 (13.8%)	PFS: 2.1 vs. 3.3 months HR: 0.98 (0.79–1.16) OS: 10.1 vs. 7.3 months HR: 0.70 (0.57–0.86) ORR: 21.1% vs. 11.4% (*p* =0.001)	OS: HR 0.53 (95% CI 0.28–1.01)
NCT02603432 JAVELIN Bladder 100 [29]	Avelumab vs. BSC (after disease control with chemo)	Maintenance/ Phase 3	700–187 (26%)	PFS: 3.7 vs. 2.0 months HR: 0.62 (0.52–0.75) OS: 21.4 vs. 14.3 months HR: 0.69 (0.56–0.86)	PFS: 2.7 vs. 2months HR: 0.85 (0.60–1.21) OS: 19.9 vs. 17.4 months HR: 0.90 (0.59–1.39)
NCT05327530 (JAVELIN BladderMedley) [35]	Avelumab monotherapy control vs. multiple experimental combos in patients progression-free after 1L platinum chemo.	Maintenance/Phase 2 (umbrella, randomized)	NA	Sacituzumabgovitecan PFS: 11.2 vs. 3.8 months HR: 0.49	NA

PFS: progression-free survival, OS: overall survival, ORR: objective response rate, NA: Not Available.

## Data Availability

No new data were created or analyzed in this study.

## Supplementary Materials

The following supporting information can be downloaded at: https://www.mdpi.com/article/10.3390/cancers18101643/s1, Table S1. Immunotherapeutic agents currently investigated or approved for upper tract urothelial carcinoma and their molecular targets.

## Abbreviations

The following abbreviations are used in this manuscript:

UTUC	Upper Tract Urothelial Carcinoma
UCB	Urothelial Carcinoma of the Bladder
RNU	Radical Nephroureterectomy
MIBC	Muscle-Invasive Bladder Cancer
OS	Overall Survival
PFS	Progression-Free Survival
EFS	Event-Free Survival
DFS	Disease-Free Survival
ORR	Objective Response Rate
HR	Hazard Ratio
pCR	Pathological Complete Response
ICIs	Immune Checkpoint Inhibitors
irAEs	immune-related Adverse Events

## References

[1] Masson-Lecomte A., Birtle A., Pradere B., Capoun O., Compérat E., Domínguez-Escrig J.L., Liedberg F., Makaroff L., Mariappan P., Moschini M. (2025). European Association of Urology Guidelines on Upper Urinary Tract Urothelial Carcinoma: Summary of the 2025 Update. Eur. Urol..

[2] Gontero P., Birtle A., Capoun O., Compérat E., Dominguez-Escrig J.L., Liedberg F., Mariappan P., Masson-Lecomte A., Mostafid H.A., Pradere B. (2024). European Association of Urology Guidelines on Non-muscle-invasive Bladder Cancer (TaT1 and Carcinoma In Situ)—A Summary of the 2024 Guidelines Update. Eur. Urol..

[3] Leow J.J., Chong Y.L., Chang S.L., Valderrama B.P., Powles T., Bellmunt J. (2021). Neoadjuvant and Adjuvant Chemotherapy for Upper Tract Urothelial Carcinoma: A 2020 Systematic Review and Meta-analysis, and Future Perspectives on Systemic Therapy. Eur. Urol..

[4] Califano G., Xylinas E. (2020). Re: Phase II Trial of Neoadjuvant Systemic Chemotherapy Followed by Extirpative Surgery in Patients with High Grade Upper Tract Urothelial Carcinoma. Eur. Urol..

[5] Collà Ruvolo C., Nocera L., Stolzenbach L.F., Wenzel M., Cucchiara V., Tian Z., Shariat S.F., Saad F., Longo N., Montorsi F. (2021). Incidence and Survival Rates of Contemporary Patients with Invasive Upper Tract Urothelial Carcinoma. Eur. Urol. Oncol..

[6] Evmorfopoulos K., Mitrakas L., Karathanasis A., Zachos I., Tzortzis V., Vlachostergios P.J. (2023). Upper Tract Urothelial Carcinoma: A Rare Malignancy with Distinct Immuno-Genomic Features in the Era of Precision-Based Therapies. Biomedicines.

[7] Califano G., Ouzaid I., Verze P., Hermieu J.F., Mirone V., Xylinas E. (2021). Immune checkpoint inhibition in upper tract urothelial carcinoma. World J. Urol..

[8] van der Heijden A.G., Bruins H.M., Carrion A., Cathomas R., Compérat E., Dimitropoulos K., Efstathiou J.A., Fietkau R., Kailavasan M., Lorch A. (2025). European Association of Urology Guidelines on Muscle-invasive and Metastatic Bladder Cancer: Summary of the 2025 Guidelines. Eur. Urol..

[9] Necchi A., Martini A., Raggi D., Cucchiara V., Colecchia M., Lucianò R., Villa L., Mazzone E., Basile G., Scuderi S. (2022). A feasibility study of preoperative pembrolizumab before radical nephroureterectomy in patients with high-risk, upper tract urothelial carcinoma: PURE-02. Urol. Oncol..

[10] Birtle A., Johnson M., Chester J., Jones R., Dolling D., Bryan R.T., Harris C., Winterbottom A., Blacker A., Catto J.W.F. (2020). Adjuvant chemotherapy in upper tract urothelial carcinoma (the POUT trial): A phase 3, open-label, randomised controlled trial. Lancet.

[11] Bellmunt J., Hussain M., Gschwend J.E., Albers P., Oudard S., Castellano D., Daneshmand S., Nishiyama H., Majchrowicz M., Degaonkar V. (2021). Adjuvant atezolizumab versus observation in muscle-invasive urothelial carcinoma (IMvigor010): A multicentre, open-label, randomised, phase 3 trial. Lancet Oncol..

[12] Bajorin D.F., Witjes J.A., Gschwend J.E., Schenker M., Valderrama B.P., Tomita Y., Bamias A., Lebret T., Shariat S.F., Park S.H. (2021). Adjuvant Nivolumab versus Placebo in Muscle-Invasive Urothelial Carcinoma. N. Engl. J. Med..

[13] Galsky M.D., Gschwend J.E., Milowsky M.I., Schenker M., Valderrama B.P., Tomita Y., Bamias A., Lebret T., Shariat S.F., Park S.H. (2026). Adjuvant nivolumab versus placebo for high-risk muscle-invasive urothelial carcinoma: 5-year efficacy and ctDNA results from CheckMate 274. Ann. Oncol..

[14] Apolo A.B., Ballman K.V., Sonpavde G., Berg S., Kim W.Y., Parikh R., Teo M.Y., Sweis R.F., Geynisman D.M., Grivas P. (2025). Adjuvant Pembrolizumab versus Observation in Muscle-Invasive Urothelial Carcinoma. N. Engl. J. Med..

[15] Laukhtina E., Sari Motlagh R., Mori K., Katayama S., Rajwa P., Yanagisawa T., Quhal F., Mostafaei H., Grossmann N.C., König F. (2022). Chemotherapy is superior to checkpoint inhibitors after radical surgery for urothelial carcinoma: A systematic review and network meta-analysis of oncologic and toxicity outcomes. Crit. Rev. Oncol. Hematol..

[16] Galsky M.D., Hoimes C.J., Necchi A., Shore N., Witjes J.A., Steinberg G., Bedke J., Nishiyama H., Fang X., Kataria R. (2021). Perioperative pembrolizumab therapy in muscle-invasive bladder cancer: Phase III KEYNOTE-866 and KEYNOTE-905/EV-303. Future Oncol..

[17] Galsky M.D., Valderrama B.P., Maruzzo M., Font Pous A., Ciuleanu T.E., Chatzkel J.A., Koie T., Hoimes C.J., Puente J., Zakharia Y. (2026). Neoadjuvant and adjuvant enfortumab vedotin plus pembrolizumab for participants with muscle-invasive bladder cancer eligible for cisplatin: Randomized, open-label, phase 3 KEYNOTE-B15 study. J. Clin. Oncol..

[18] Powles T., Catto J.W.F., Galsky M.D., Al-Ahmadie H., Meeks J.J., Nishiyama H., Vu T.Q., Antonuzzo L., Wiechno P., Atduev V. (2024). Perioperative Durvalumab with Neoadjuvant Chemotherapy in Operable Bladder Cancer. N. Engl. J. Med..

[19] Hoimes C.J., Flaig T.W., Milowsky M.I., Friedlander T.W., Bilen M.A., Gupta S., Srinivas S., Merchan J.R., McKay R.R., Petrylak D.P. (2023). Enfortumab Vedotin Plus Pembrolizumab in Previously Untreated Advanced Urothelial Cancer. J. Clin. Oncol..

[20] Powles T., Valderrama B.P., Gupta S., Bedke J., Kikuchi E., Hoffman-Censits J., Iyer G., Vulsteke C., Park S.H., Shin S.J. (2024). Enfortumab Vedotin and Pembrolizumab in Untreated Advanced Urothelial Cancer. N. Engl. J. Med..

[21] van der Heijden M.S., Sonpavde G., Powles T., Necchi A., Burotto M., Schenker M., Sade J.P., Bamias A., Beuzeboc P., Bedke J. (2023). Nivolumab plus Gemcitabine-Cisplatin in Advanced Urothelial Carcinoma. N. Engl. J. Med..

[22] Tomita Y., Ye D.W., Fujii A., Takeuchi N. (2025). Nivolumab plus gemcitabine-cisplatin for previously untreated unresectable or metastatic urothelial carcinoma: An Asian subgroup analysis from the global phase 3 CheckMate 901 trial. Urol. Oncol..

[23] Wheatley D.A., Berardi R., Climent Duran M.A., Tomiak A., Greystoke A.P., Joshua A.M., Arkenau H.T., Géczi L., Corbacho J.G., Paz-Ares L.G. (2024). First-line Avelumab plus Chemotherapy in Patients with Advanced Solid Tumors: Results from the Phase Ib/II JAVELIN Chemotherapy Medley Study. Cancer Res. Commun..

[24] Powles T., Csőszi T., Özgüroğlu M., Matsubara N., Géczi L., Cheng S.Y., Fradet Y., Oudard S., Vulsteke C., Morales Barrera R. (2021). Pembrolizumab alone or combined with chemotherapy versus chemotherapy as first-line therapy for advanced urothelial carcinoma (KEYNOTE-361): A randomised, open-label, phase 3 trial. Lancet Oncol..

[25] Escobar D., Wang C., Suboc N., D’Souza A., Tulpule V. (2025). Diagnosis and Management of Upper Tract Urothelial Carcinoma: A Review. Cancers.

[26] Rosenberg J.E., Galsky M.D., Powles T., Petrylak D.P., Bellmunt J., Loriot Y., Necchi A., Hoffman-Censits J., Perez-Gracia J.L., van der Heijden M.S. (2024). Atezolizumab monotherapy for metastatic urothelial carcinoma: Final analysis from the phase II IMvigor210 trial. ESMO Open.

[27] Galsky M.D., Arija J.Á.A., Bamias A., Davis I.D., De Santis M., Kikuchi E., Garcia-Del-Muro X., De Giorgi U., Mencinger M., Izumi K. (2020). Atezolizumab with or without chemotherapy in metastatic urothelial cancer (IMvigor130): A multicentre, randomised, placebo-controlled phase 3 trial. Lancet.

[28] Balar A.V., Castellano D., O’Donnell P.H., Grivas P., Vuky J., Powles T., Plimack E.R., Hahn N.M., de Wit R., Pang L. (2017). First-line pembrolizumab in cisplatin-ineligible patients with locally advanced and unresectable or metastatic urothelial cancer (KEYNOTE-052): A multicentre, single-arm, phase 2 study. Lancet Oncol..

[29] Powles T., Park S.H., Caserta C., Valderrama B.P., Gurney H., Ullén A., Loriot Y., Sridhar S.S., Sternberg C.N., Bellmunt J. (2023). Avelumab First-Line Maintenance for Advanced Urothelial Carcinoma: Results From the JAVELIN Bladder 100 Trial After ≥2 Years of Follow-Up. J. Clin. Oncol..

[30] Powles T., van der Heijden M.S., Castellano D., Galsky M.D., Loriot Y., Petrylak D.P., Ogawa O., Park S.H., Lee J.L., De Giorgi U. (2020). Durvalumab alone and durvalumab plus tremelimumab versus chemotherapy in previously untreated patients with unresectable, locally advanced or metastatic urothelial carcinoma (DANUBE): A randomised, open-label, multicentre, phase 3 trial. Lancet Oncol..

[31] Powles T., Park S.H., Voog E., Caserta C., Valderrama B.P., Gurney H., Kalofonos H., Radulović S., Demey W., Ullén A. (2020). Avelumab Maintenance Therapy for Advanced or Metastatic Urothelial Carcinoma. N. Engl. J. Med..

[32] Ciciriello F., Pezzicoli G., Biasi A., D’Addario C., Salonne F., Porta C., Rizzo M. (2025). HER2-Positive Urothelial Carcinoma: Current Evidence on Targeted Agents and Immunotherapy-Based Combinations. Target. Oncol..

[33] Powles T., Assaf Z.J., Davarpanah N., Banchereau R., Szabados B.E., Yuen K.C., Grivas P., Hussain M., Oudard S., Gschwend J.E. (2021). ctDNA guiding adjuvant immunotherapy in urothelial carcinoma. Nature.

[34] Grivas P., Park S.H., Voog E., Caserta C., Gurney H., Bellmunt J., Kalofonos H., Ullén A., Loriot Y., Sridhar S.S. (2023). Avelumab First-line Maintenance Therapy for Advanced Urothelial Carcinoma: Comprehensive Clinical Subgroup Analyses from the JAVELIN Bladder 100 Phase 3 Trial. Eur. Urol..

[35] Hoffman-Censits J., Grivas P., Powles T., Hawley J., Tyroller K., Seeberger S., Guenther S., Jacob N., Mehr K.T., Hahn N.M. (2024). The JAVELIN Bladder Medley trial: Avelumab-based combinations as first-line maintenance in advanced urothelial carcinoma. Future Oncol..

[36] Hoffman-Censits J., Tsiatas M., Chang P.M., Kim M., Antonuzzo L., Shin S.J., Gakis G., Blais N., Kim S.H., Smith A. (2025). Avelumab plus sacituzumab govitecan versus avelumab monotherapy as first-line maintenance treatment in patients with advanced urothelial carcinoma: JAVELIN Bladder Medley interim analysis. Ann. Oncol..

[37] Bellmunt J., de Wit R., Vaughn D.J., Fradet Y., Lee J.L., Fong L., Vogelzang N.J., Climent M.A., Petrylak D.P., Choueiri T.K. (2017). Pembrolizumab as Second-Line Therapy for Advanced Urothelial Carcinoma. N. Engl. J. Med..

[38] Sharma P., Retz M., Siefker-Radtke A., Baron A., Necchi A., Bedke J., Plimack E.R., Vaena D., Grimm M.O., Bracarda S. (2017). Nivolumab in metastatic urothelial carcinoma after platinum therapy (CheckMate 275): A multicentre, single-arm, phase 2 trial. Lancet Oncol..

[39] Rosenberg J.E., Hoffman-Censits J., Powles T., van der Heijden M.S., Balar A.V., Necchi A., Dawson N., O’Donnell P.H., Balmanoukian A., Loriot Y. (2016). Atezolizumab in patients with locally advanced and metastatic urothelial carcinoma who have progressed following treatment with platinum-based chemotherapy: A single-arm, multicentre, phase 2 trial. Lancet.

[40] Powles T., Durán I., van der Heijden M.S., Loriot Y., Vogelzang N.J., De Giorgi U., Oudard S., Retz M.M., Castellano D., Bamias A. (2018). Atezolizumab versus chemotherapy in patients with platinum-treated locally advanced or metastatic urothelial carcinoma (IMvigor211): A multicentre, open-label, phase 3 randomised controlled trial. Lancet.

[41] van der Heijden M.S., Loriot Y., Durán I., Ravaud A., Retz M., Vogelzang N.J., Nelson B., Wang J., Shen X., Powles T. (2021). Atezolizumab Versus Chemotherapy in Patients with Platinum-treated Locally Advanced or Metastatic Urothelial Carcinoma: A Long-term Overall Survival and Safety Update from the Phase 3 IMvigor211 Clinical Trial. Eur. Urol..

[42] Muthigi A., George A.K., Brancato S.J., Agarwal P.K. (2016). Novel immunotherapeutic approaches to the treatment of urothelial carcinoma. Ther. Adv. Urol..

[43] Esagian S.M., Khaki A.R., Diamantopoulos L.N., Carril-Ajuria L., Castellano D., De Kouchkovsky I., Park J.J., Alva A., Bilen M.A., Stewart T.F. (2021). Immune checkpoint inhibitors in advanced upper and lower tract urothelial carcinoma: A comparison of outcomes. BJU Int..

[44] Grimm M.O., Bex A., De Santis M., Ljungberg B., Catto J.W.F., Rouprêt M., Hussain S.A., Bellmunt J., Powles T., Wirth M. (2019). Safe Use of Immune Checkpoint Inhibitors in the Multidisciplinary Management of Urological Cancer: The European Association of Urology Position in 2019. Eur. Urol..

[45] Su R., Chen Z., Hong D., Jiang S., Yuan Y., Cai X., Hu H., Fu C., Huang Z., Wang Z. (2023). Effectiveness and safety of immune checkpoint inhibitor monotherapy in advanced upper tract urothelial carcinoma: A multicenter, retrospective, real-world study. Cancer Med..

[46] Khoja L., Day D., Wei-Wu Chen T., Siu L.L., Hansen A.R. (2017). Tumour- and class-specific patterns of immune-related adverse events of immune checkpoint inhibitors: A systematic review. Ann. Oncol..

[47] Spain L., Diem S., Larkin J. (2016). Management of toxicities of immune checkpoint inhibitors. Cancer Treat. Rev..

[48] Wang P.F., Chen Y., Song S.Y., Wang T.J., Ji W.J., Li S.W., Liu N., Yan C.X. (2017). Immune-Related Adverse Events Associated with Anti-PD-1/PD-L1 Treatment for Malignancies: A Meta-Analysis. Front. Pharmacol..

[49] Wu Z., Chen Q., Qu L., Li M., Wang L., Mir M.C., Carbonara U., Pandolfo S.D., Black P.C., Paul A.K. (2022). Adverse Events of Immune Checkpoint Inhibitors Therapy for Urologic Cancer Patients in Clinical Trials: A Collaborative Systematic Review and Meta-Analysis. Eur. Urol..

[50] Zhao D., Long X., Fan H., Wang J. (2022). Effects of hepatic or renal impairment on the pharmacokinetics of immune checkpoint inhibitors. Am. J. Cancer Res..

[51] Miao J., Sise M.E., Herrmann S.M. (2022). Immune checkpoint inhibitor related nephrotoxicity: Advances in clinicopathologic features, noninvasive approaches, and therapeutic strategy and rechallenge. Front. Nephrol..

[52] Tiu B.C., Strohbehn I.A., Zhao S., Ouyang T., Hanna P., Wang Q., Gupta S., Leaf D.E., Reynolds K.L., Sise M.E. (2023). Safety of Immune Checkpoint Inhibitors in Patients with Advanced Chronic Kidney Disease: A Retrospective Cohort Study. Oncologist.

[53] Jose Manzanera Escribano M., Morales Ruiz E., Odriozola Grijalba M., Gutierrez Martinez E., Rodriguez Antolin A., Praga Terente M. (2007). Acute renal failure due to interstitial nephritis after intravesical instillation of BCG. Clin. Exp. Nephrol..

[54] Bajramovic S., Alic J., Skopljak E., Chikha A., Vesnic S., Smajilbegovic V., Aganovic D. (2020). Renal Tuberculosis Following Intravesical Bacillus Calmette-Guérin (BCG) Immunotherapy for the Treatment of Bladder Cancer. Med. Arch..

[55] Fradet Y., Bellmunt J., Vaughn D.J., Lee J.L., Fong L., Vogelzang N.J., Climent M.A., Petrylak D.P., Choueiri T.K., Necchi A. (2019). Randomized phase III KEYNOTE-045 trial of pembrolizumab versus paclitaxel, docetaxel, or vinflunine in recurrent advanced urothelial cancer: Results of >2 years of follow-up. Ann. Oncol..

[56] Crist M., Iyer G., Hsu M., Huang W.C., Balar A.V. (2019). Pembrolizumab in the treatment of locally advanced or metastatic urothelial carcinoma: Clinical trial evidence and experience. Ther. Adv. Urol..

[57] Angelopoulos P., Markopoulos T., Lazarou L., Skolarikos A., Stamatakos P., Papadopoulos G.I., Fragkoulis C., Ntoumas K., Moulavasilis N., Levis P. (2024). Retrospective, Non-Interventional, Multicenter Study on the Effectiveness and Safety of Intravesical Bacillus Calmette-Guerin in Patients with Non-Muscle-Invasive Bladder Cancer: Real-World Experience from Six Hospital Centers in Greece. Curr. Oncol..

[58] Trisal S.R., Low G., Pathan F., Gangadharan Komala M. (2023). Kidney Adverse Events Associated with Immune Checkpoint Inhibitor Therapy: A Systematic Review and Bayesian Network Meta-Analysis. Clin. J. Am. Soc. Nephrol..

[59] Righini M., Mollica V., Rizzo A., La Manna G., Massari F. (2022). Renal Toxicities in Cancer Patients Receiving Immune-Checkpoint Inhibitors: A Meta-Analysis. J. Clin. Med..

