# Supplementary material for: Immunotherapy for Upper Tract Urothelial Carcinomas: A 2026 Update
Source: Cancers (Basel). 2026 May 19;18(10):1643. doi: 10.3390/cancers18101643 (PMC13204836; doi:10.3390/cancers18101643)
Supplement: Supplementary file 1 [file cancers-18-01643-s001.zip › cancers-4308480-supplementary.pdf]

Supplementary Table S1. Immunotherapeutic agents currently investigated or approved for upper tract urothelial carcinoma and their molecular targets.

**A. Immune checkpoint inhibitors**		
**Drug**	**Class**	**Molecular Target**
Pembrolizumab	Monoclonal Antibody	PD-1
Nivolumab	Monoclonal Antibody	PD-1
Atezolizumab	Monoclonal Antibody	PD-L1
Avelumab	Monoclonal Antibody	PD-L1
Durvalumab	Monoclonal Antibody	PD-L1
**B. Antibody–drug conjugate**		
**Drug**	**Target**	**Cytotoxic Component**
Enfortumab vedotin	Nectin-4	Monomethyl auristatin E
Sacituzumab govitecan	Trop-2	SN-38
**C. Combination Therapies**		
Combination regimens	PD-1/PD-L1 blockade + chemotherapy	
